# PhoglyStruct: Prediction of phosphoglycerylated lysine residues using structural properties of amino acids

**DOI:** 10.1038/s41598-018-36203-8

**Published:** 2018-12-18

**Authors:** Abel Chandra, Alok Sharma, Abdollah Dehzangi, Shoba Ranganathan, Anjeela Jokhan, Kuo-Chen Chou, Tatsuhiko Tsunoda

**Affiliations:** 1 Institute for Integrated and Intelligent Systems, Griffith University, Brisbane, QLD-4111 Australia; 2 Department of Medical Science Mathematics, Medical Research Institute, Tokyo Medical and Dental University, Tokyo, 113-8510 Japan; 3 Laboratory for Medical Science Mathematics, RIKEN Center for Integrative Medical Sciences, Yokohama, 230-0045 Kanagawa Japan; 4 Department of Computer Science, Morgan State University, Baltimore, Maryland USA; 5 School of Engineering and Physics, Faculty of Science Technology and Environment, University of the South Pacific, Suva, Fiji; 6 Department of Molecular Sciences, Macquarie University, Sydney, NSW 2109 Australia; 7 Faculty of Science Technology and Environment, University of the South Pacific, Suva, Fiji; 8 The Gordon Life Science Institute, Boston, MA 02478 USA; 9 CREST, JST, Tokyo, 113-8510 Japan

## Abstract

The biological process known as post-translational modification (PTM) contributes to diversifying the proteome hence affecting many aspects of normal cell biology and pathogenesis. There have been many recently reported PTMs, but lysine phosphoglycerylation has emerged as the most recent subject of interest. Despite a large number of proteins being sequenced, the experimental method for detection of phosphoglycerylated residues remains an expensive, time-consuming and inefficient endeavor in the post-genomic era. Instead, the computational methods are being proposed for accurately predicting phosphoglycerylated lysines. Though a number of predictors are available, performance in detecting phosphoglycerylated lysine residues is still limited. In this paper, we propose a new predictor called PhoglyStruct that utilizes structural information of amino acids alongside a multilayer perceptron classifier for predicting phosphoglycerylated and non-phosphoglycerylated lysine residues. For the experiment, we located phosphoglycerylated and non-phosphoglycerylated lysines in our employed benchmark. We then derived and integrated properties such as accessible surface area, backbone torsion angles, and local structure conformations. PhoglyStruct showed significant improvement in the ability to detect phosphoglycerylated residues from non-phosphoglycerylated ones when compared to previous predictors. The sensitivity, specificity, accuracy, Mathews correlation coefficient and AUC were 0.8542, 0.7597, 0.7834, 0.5468 and 0.8077, respectively. The data and Matlab/Octave software packages are available at https://github.com/abelavit/PhoglyStruct.

## Introduction

Post-translational modifications (PTMs) play a very crucial role in cell functions and biological processes as well as regulating plasticity and dynamics. The emergence of high-throughput proteomics efforts regarding the study of site-specific PTM and protein modifying enzymes has caused the stir of interest in modifications across various organisms^1^. Out of the 20 amino acids in the genetic code, lysine is the most heavily modified^2,3^. In the literature, it is seen that lysine residues are prone to covalent modifications such as acetyl^4^, glycosyl^5^, succinyl^6^, crotonyl^7^, methyl^8^, propionyl^9^ and pupyl^10^. There are a variety of human diseases associated with the modification of amino acids and their regulatory enzymes and these include heart disease, multiple sclerosis, celiac disease, rheumatoid arthritis, and neurodegenerative disorders^11–14^.

Phosphoglycerylation is a newly identified non-enzymatic lysine modification found in mouse liver as well as in human cells^15,16^. This chemical modification is linked to glycolytic pathways and glucose metabolism resulting in the high association with cardiovascular diseases such as heart failure^17,18^. Phosphoglycerylation is a dynamic and reversible biochemical process whereby a primary glycolytic intermediate (1,3-BPG) and lysine residue react to form 3-phosphoglyceryl-lysine (pgK)^16^. 3-phosphoglyceryl-lysine modifications hinder glycolytic enzymes, and it accumulates on these enzymes for cells exposed to high glucose creating a potential feedback mechanism that leads to the buildup and redirection of glycolytic intermediates to other biosynthetic pathways. Since there is little known about this PTM, identification, and analysis of its functional aspects are vital for understanding its selectivity mechanism and regulatory roles for the diagnosis and treatment of individuals affected.

The method of pure experimental procedure in laboratories such as mass spectrometry for identifying phosphoglycerylated sites in protein sequences is inefficient, time-consuming and expensive^19–21^ hence there is a growing interest in the development of computationally based predictors to identify them^22–35^. The ability for computational tools to predict phosphoglycerylated sites has demonstrated itself to be an absolute necessity for dealing with the identification of the PTM over the experimental procedure. There have been several studies proposed for this purpose. Phogly-PseAAC uses a KNN-based predictor based on the pseudo amino acid composition features^36^. CKSAAP_PhoglySite utilizes the composition of k-spaced amino acid pairs (CKSAAP) and Chou’s PseAAC. It employs weight assignment to deal with class imbalance and uses a fuzzy support vector machine for prediction^15^. The PhoglyPred uses the sequence information derived from the increment of k-mer diversity, the position-specific propensity of k-space dipeptide and the modified composition of k-space amino acid pairs with selected physicochemical attributes. To deal with class imbalance, it also utilizes weight assignment to the training samples with SVM classifier^37^. The most recent work to identify lysine phosphoglycerylation is iPGK-PseAAC^38^ which uses four tiers of amino acid pairwise coupling information into the general PseAAC with SVM as the operation engine.

In this paper, we propose a new predictor called PhoglyStruct which examines a comprehensive set of structural properties to distinguish between phosphoglycerylated and non-phosphoglycerylated lysine residues. For classification, the PhoglyStruct predictor employs a multilayer perceptron classifier. In this work, we utilized 91 proteins with experimentally detected phosphoglycerylated residues and obtained the properties namely accessible surface area (ASA), the probability of amino acid contribution to local structure conformations (coil, strand, and helix) and finally backbone torsion angles. After the eight properties were obtained for each amino acid in the protein sequences, a test was conducted to find the number of upstream and downstream of each lysine residue that resulted in the highest performance. The ±2 residue window proved to be a promising segment size which yielded the highest geometric mean (G-Mean) when the segment size of 15 and below were assessed (see Supplementary Material 1). Hence the feature vector of 8 properties for each amino acid was created for phosphoglycerylated and non-phosphoglycerylated lysine residues by considering a stretch of a sequence comprising 2 upstream and 2 downstream amino acid and the lysine itself in the middle. Due to a considerable number of non-phosphoglycerylated lysine residues compared to phosphoglycerylated residues, we implemented the k-nearest neighbors cleaning treatment^19^. This procedure enables us to construct our benchmark dataset by resolving the data imbalance issue. This benchmark dataset was then used to train the multilayer perceptron for classification purposes. The backward elimination scheme^39^ was used to select the useful properties and the performance was evaluated using the 10-fold cross-validation procedure. Furthermore, we compared PhoglyStruct with a simpler set of features for the same 10-fold cross-validation set and the comparison showed the use of structural properties of PhoglyStruct to be advantageous (see Supplementary Material 2). We found that PhoglyStruct showed considerable improvement in the ability to detect phosphoglycerylated residues from non-phosphoglycerylated residues over the previous methods. PhoglyStruct has been able to successfully classify phosphoglycerylated residues with 0.8542 sensitivity, 0.7597 specificity, 0.8022 G-Mean, 0.7834 accuracy, 0.5468 Mathews correlation coefficient, 0.6603 F-Measure, and 0.8077 area under the ROC curve (AUC).

## Materials and Methods

The following sections describe the construction of benchmark data and the selection of properties for a segment of amino acids corresponding to the lysine residues.

### Benchmark dataset

In this work, we used the CPLM repository (http://cplm.biocuckoo.org) to construct our phosphoglycerylation dataset. It is a database containing experimentally identified PTM sites for a number of protein lysine modifications. For lysine phosphoglycerylation, we filtered out the protein sequences by removing those sequences with ≥40% sequential similarities using the CD-HIT tool^40^. In the 91 resulting protein sequences that we retrieved, there were a total of 3360 lysine residues and of these lysine residues, 111 were phosphoglycerylated. The negative samples are filtered out to reduce data imbalance thereby giving phosphoglycerylation benchmark dataset.

The number of phosphoglycerylated sites (positive set) was just 111 compared to 3249 non-phosphoglycerylated sites (negative set). This highly imbalanced sets result in a ratio of 1:29 which most likely would lead to a completely biased classification result. Dealing with class imbalance is an essential step in classification problems. At this stage, to avoid bias during the classification stage, we removed the redundant instances. To do this, we adopted the k-nearest neighbor strategy which has been quite commonly used in the literature^19,21,41–43^. Here, we remove the redundant negative samples using the k-nearest neighbors cleaning treatment by firstly calculating the Euclidean distance between each sample in the dataset. The initial number of neighbors, *k*, was computed by dividing the number of negative samples with positive samples. So the initial value of *k* used was 29 to reduce the imbalance in the classes. The idea is to remove a negative instance when one of its 29 nearest neighbors is a positive instance, based on the Euclidean distances. It was found that the class imbalance remained high after the first filtering procedure with *k* = 29. The threshold was increased further until we noticed the negative set was thrice the positive set. The *k* value of 69 reduced the number of negative samples to 337 from 3249. Hence a negative sample was removed when one of its 69 neighbors was a positive sample. While dealing with the class imbalance issue, the positive instances remained the same. As a result, we had 337 negative samples and 111 positive samples which made up the benchmark dataset. These sets were then used to carry out 10-fold cross-validation and evaluate the performance of our predictor PhoglyStruct.

### Amino acid characteristics

We obtained eight properties corresponding to the accessible surface area, backbone torsion angles, and secondary structure for each amino acid in the protein sequences. These characteristics were obtained using the newly developed toolbox SPIDER2^44^. The SPIDER2 toolbox is known to achieve good results regarding the prediction of the accessible surface area^45–47^, secondary structure^48,49^ and backbone torsion angles^45,50^ in proteins. It has also been reported for successful extraction of structural properties of proteins for sequence-based binding sites prediction^51,52^. These features are considered important source to provide information about the local interaction of amino acids along the protein sequence. Also, they have been used in different studies to tackle different problems in protein science and attained promising results^53–56^. The subsequent sections below discuss these structural properties.

#### Accessible surface area

The estimate of the accessible area of an amino acid to a solvent in the 3D configuration of a protein is given by ASA^57,58^. Hence, essential information on the protein structure is revealed by the predicted ASA of individual amino acids. SPIDER2 is executed on each protein sequence for ASA computation, and the resultant estimated value for each amino acid is obtained. It is worthwhile to mention that SPIDER2 uses only the primary sequence of proteins, so the prediction is entirely based on sequence information.

#### Secondary structure

Secondary structure gives the information on the local 3D structure of proteins. For each amino acid, the predicted secondary structure provides a discrete output of its contribution to one of the three defined local structures of a protein which are coil, strand, and helix. The secondary structure, therefore, provides vital information in understanding the protein’s general 3D configuration. We again run SPIDER2 for each protein to predict the probability of each amino acid’s conformation to the three local structures namely: coil (C) (*pc*), strand (E) (*pe*) and helix (H) (*ph*). The output of SPIDER2 is an *L* × 3 matrix, where *L* represents the length of the protein and the three columns represent the transitional probabilities to the three secondary structure conformations. This matrix is called SS*pre* for simplicity.

#### Local backbone angles

**Figure 1:**
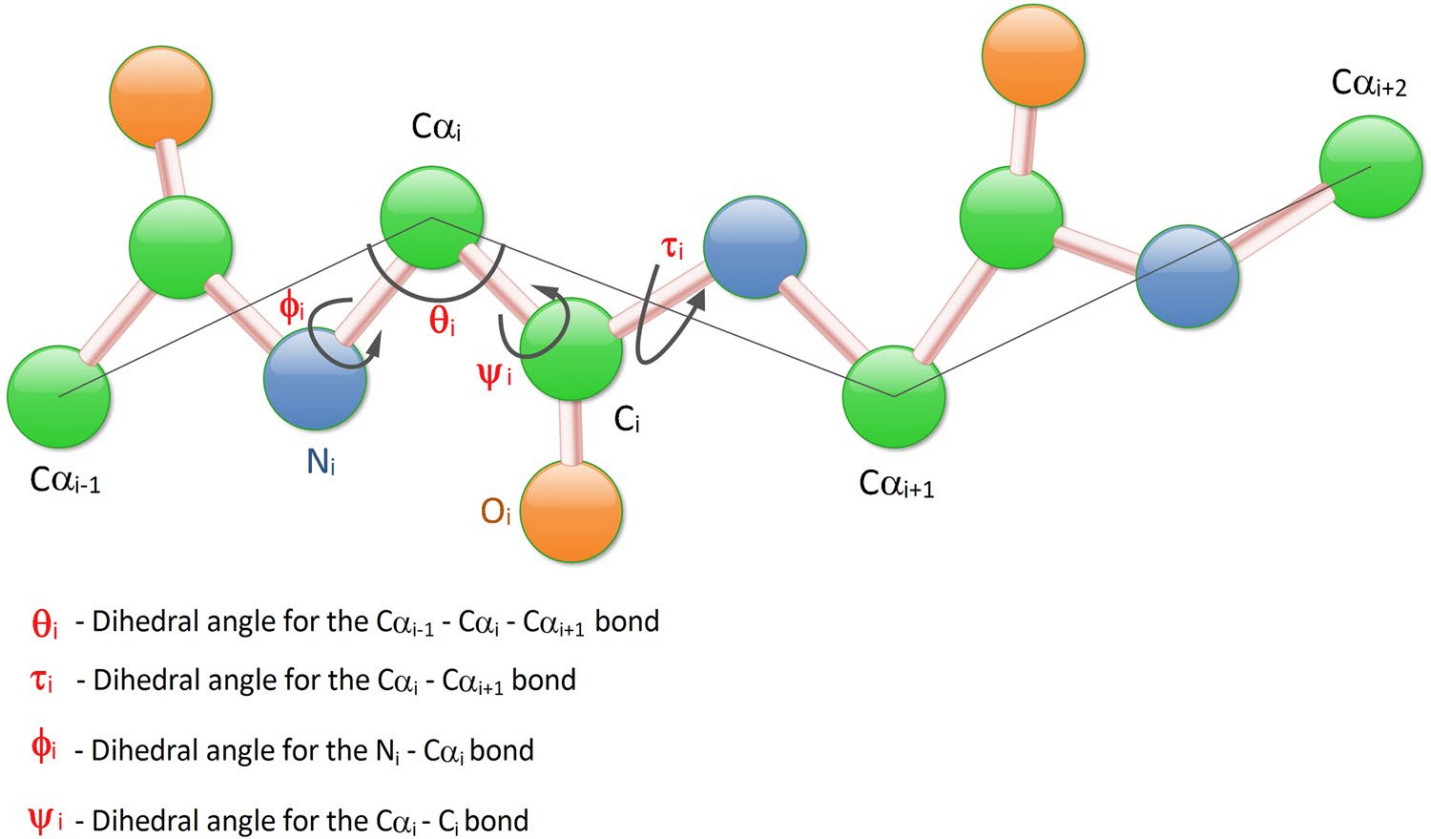
Illustration of torsion angles associated with the protein backbone.

Torsion angles, which are the angles between neighboring amino acids, complements ASA as well as the predicted secondary structure by providing important, continuous information about the local structure of amino acids^50^. The predicted Backbone torsion angles, *ϕ*, and *ψ*, of the local amino acid, provides information regarding the continuous representation of its interaction along the protein backbone^59,60^. For a given amino acid, the angle ϕ_i_ is the dihedral angle for the N_i_ - Cα_i_ bond while ψ_i_ is the angle rotated about Cα_i_ - C_i_ bond. There has been an inclusion of two new angles in recent studies which are based on dihedral angles *θ* (angle between three Cα atoms Cα_i−1_ - Cα_i_ - Cα_i+1_) and *τ* (rotated about Cα_i_ - Cα_i+1_ bond)^45^, as depicted in Fig. 1. Thus we run the SPIDER2 toolbox to obtain the four angles, and the result is four different numerical vectors *ϕ, ψ, θ*, and *τ*.

### Feature extraction technique

**Figure 2:**
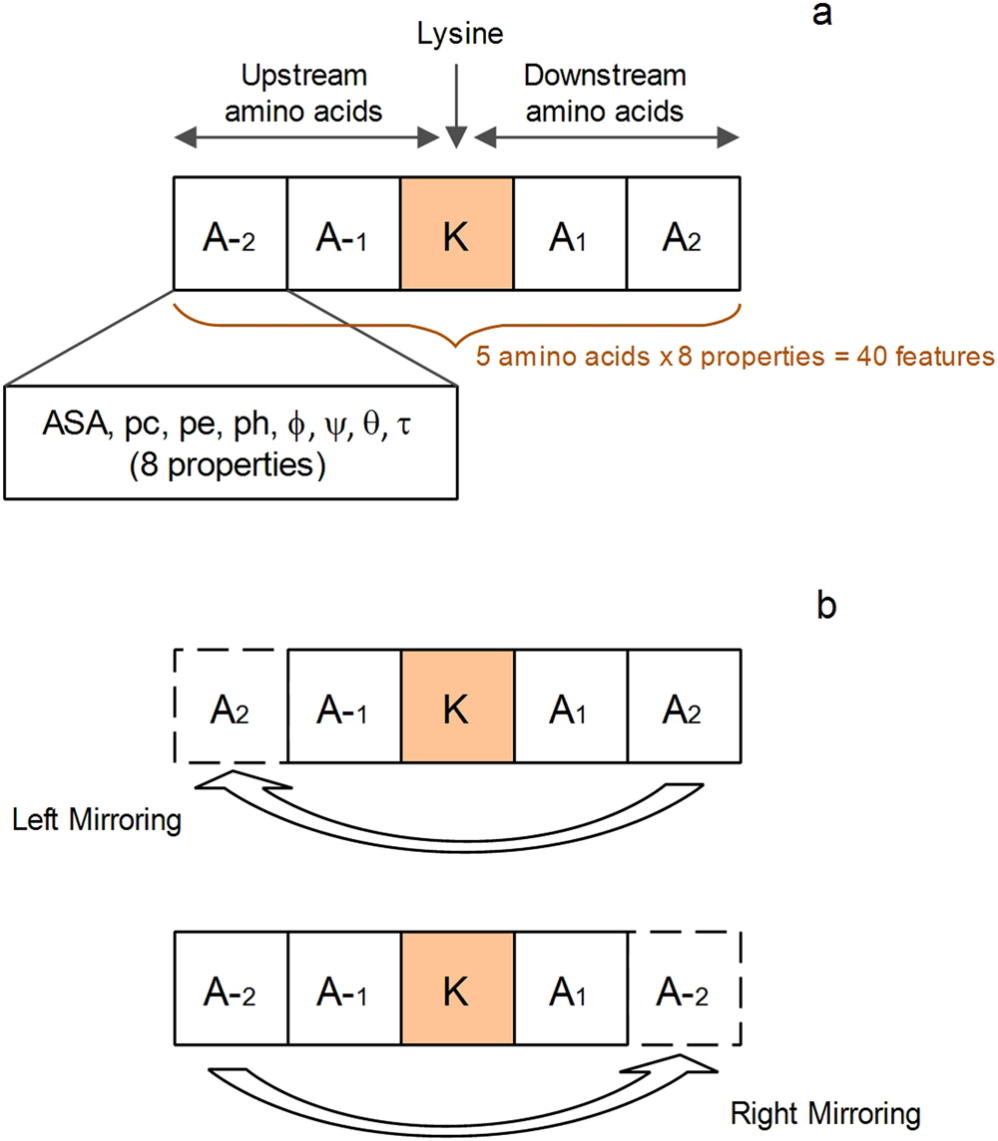
Illustration of the arrangement of neighboring amino acids to the lysine residue. (**a**) Lysine site with sufficient upstream and downstream amino acids. (**b**) Lysine site with inadequate amino acids. Left mirroring for inadequate upstream and right mirroring for insufficient downstream amino acids.

Here we will discuss the feature extraction method for each of the lysine residues. The 2 upstream and 2 downstream amino acids neighboring the lysine residue *K* is indicated in Fig. 2a. For the cases where the lysine residue did not have two neighboring amino acids, either upstream or downstream, the missing amino acids were created using the mirror effect^53^ as shown in Fig. 2b.

The peptide sequence comprising 2 upstream and 2 downstream amino acids, including lysine residue *K* at the center, can be expressed as:

1 $$P=[{A}_{-2},\,{A}_{-1},\,K,\,{A}_{1},\,{A}_{2}]$$

where *A*_*n*_ (for 1 ≤ n ≤ 2) is referred to as the downstream amino acids while *A*−_*n*_ (for 1 ≤ n ≤ 2) as the upstream amino acids. It can be deduced from equation (1) that a total of 5 amino acids, including lysine *K*, represent a lysine residue. A peptide representing a lysine residue has a class label y, where y = {0, 1}, which are experimentally confirmed labels. A label y = 1 indicates a phosphoglycerylated lysine residue whereas a label y = 0 describes a non-phosphoglycerylated residue. Moreover, each amino acid in peptide *P* is described by the structural properties as denoted by equation (2):

2 $${\rm{A}}=\{ASA,\,pc,\,pe,\,ph,\,\varphi ,\,\psi ,\,\theta ,\,\tau \}$$

A total of 8 properties (*ASA, pc, pe, ph, ϕ, ψ*, *θ*, and *τ*) are used to define a single amino acid. These properties are numeric, so each feature consists of a single value. Therefore, each peptide *P* composed of 5 amino acids is described by 40 (5 amino acids × 8) features.

### Multilayer Perceptron

**Figure 3:**
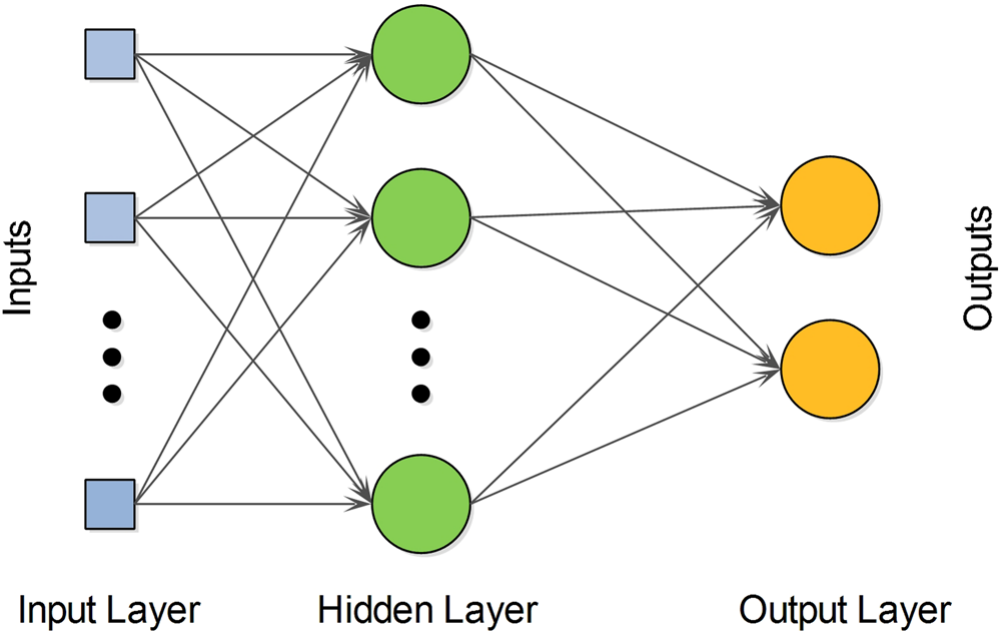
An architectural representation of the multilayer perceptron.

A Multilayer Perceptron network has three main components namely the input layer, a hidden layer, and an output layer. Input signals to the network propagate layer-by-layer. Despite the disadvantage of tuning a number of parameters such as the number of hidden neurons, it can learn highly non-linear models. The network computes an output by mapping the weighted combination of inputs through its hidden layer of nodes using a nonlinear activation function. In this work, we utilized the Weka software to generate the MLP with sigmoid function^61^. The number of nodes in the hidden layer was set to ‘a’ ((number of attributes + number of classes)/2), learning rate to 0.3, and momentum to 0.2. An architectural representation of the multilayer perceptron is shown in Fig. 3.

### Feature Selection Scheme

We have used a successive feature selection (SFS) technique to rank and select amino acid properties, out of the eight proposed in this work, which actually contribute towards the identification of phosphoglycerylation and non-phosphoglycerylation sites. The SFS scheme utilized for this purpose is called backward elimination^39^. In this method, the group of features which belong to a property is eliminated at each successive levels from the feature set. The feature set of the removed property, which resulted in the highest average G-Mean using 10-fold cross-validation on the multilayer perceptron classifier was progressed to the next subsequent level. The elimination of a property at each of the levels causes the feature set size to reduce by 5 (values for 5 amino acids corresponding to the property) as the network is progressed. At the end of the process, we obtained the ranked properties (with top ranked written first, followed by the lesser important, up till the least important property).

### Statistical measures

The most important part in the designing of a classifier for prediction is to measure the performance of the predictor. We have estimated the performance of PhoglyStruct using the seven statistical metrics generally used in the literature^15,19,36,53,62^ which are: sensitivity, specificity, G-Mean, accuracy, Mathews correlation coefficient (MCC), F-Measure and AUC. In this work, we have employed these seven metrics in-order to determine the ability of our predictor to distinguish phosphoglycerylated from non-phosphoglycerylated lysine residues in the benchmark dataset^3^.

The sensitivity metric assesses the predictor’s ability to correctly classify the phosphoglycerylated lysine residues and ranges from a value of 0 to 1. A value of 1 indicates an accurate predictor whereas a value of 0 shows it to be inaccurate. The higher the sensitivity value, the better the predictor is at detecting phosphoglycerylated lysine residues. Sensitivity metric can be defined as:

3 $$Sensitivity=\frac{TP}{TP+FN}$$

where *TP* indicates the number of true positives, which are the number of correctly predicted phosphoglycerylated lysines and *FN* represents the number of false negatives, i.e., the number of phosphoglycerylated lysines incorrectly classified by the predictor.

Specificity metric evaluates the ability of the predictor to correctly classify the non-phosphoglycerylated lysine residues. The metric value also from ranges 0 to 1 and higher the value indicates the better the predictor is at identifying non-phosphoglycerylated lysine residues. Specificity is defined as:

4 $$Specificity=\frac{TN}{TN+FP}$$

where *TN* indicates the number of true negatives, which are the number of correctly predicted non-phosphoglycerylated lysines and *FP* represents the number of false positives, i.e., the number of non-phosphoglycerylated lysines incorrectly classified by the predictor.

The geometric mean measures the balance between the classification performance of phosphoglycerylation and non-phosphoglycerylation sites. Since the idea is to identify as much phosphoglycerylation sites as possible for a given dataset, a low G-Mean would indicate poor performance in the classification of phosphoglycerylation sites even though the non-phosphoglycerylation sites have been correctly classified. G-Mean is defined as:

5 $$G-Mean=\sqrt{Sensitivity\times Specificity}$$

Accuracy is the ability of the predictor to differentiate phosphoglycerylated lysine residues from non-phosphoglycerylated ones. This is calculated by the total number of correctly classified samples (*TN* and *TP*) upon the total number of samples (*FN*, *FP*, *TN*, and *TP)*. The metric varies between 0 (least accurate) and 1 (most accurate) and is defined as:

6 $$Accuracy=\frac{TN+TP}{FN+FP+\,TN+TP}$$

Mathews correlation coefficient^63^ is used to measure the quality of a binary (two class) classifier. It is regarded as a balanced measure as it can be used when the two classes are of different sizes. The MCC metric takes on values between −1 and 1 where a 1 indicates a perfect predictor, a 0 as average and a −1 as an inverse prediction. Mathews correlation coefficient is defined as:

7 $$Mathews\,correlation\,coefficient\,=\frac{(TN\times TP)\,-\,(FN\times FP)}{\sqrt{(TP\,+\,FP)(TP\,+\,FN)(TN\,+\,FP)(TN\,+\,FN)}}$$

The F-Measure metric is calculated when the true positives are considered to be twice as important as the other cases. F-Measure ranges from 0 to 1 where a higher value indicates the better the predictor is at identifying phosphoglycerylation sites. The metric is calculated as:

8 $$F-Measure=\frac{2\times TP}{(2\times TP)+FP+\,FN}$$

### Validation scheme

The statistical measurements are obtained through the method of cross-validation to evaluate the effectiveness of a model. In statistical prediction, the following three cross-validation methods are often used to examine a predictor for its effectiveness in practical application: independent dataset test, subsampling (or K-fold cross-validation) test, and jackknife test^64,65^. However, of the three test methods, the jackknife test is deemed the least arbitrary that can always yield a unique result for a given benchmark dataset as elaborated in^66^ and demonstrated by Eqs 28–30 therein. Accordingly, the jackknife test has been widely recognized and increasingly used by investigators to examine the quality of various predictors^67–70^. However, to reduce the computational time, we adopted the 10-fold cross-validation in this study as done by many investigators. The 10-fold cross-validation scheme was applied as follows:

1. Divide the samples into ten folds of roughly equal sizes.
2. Use one fold for validation while the other nine for training.
3. Optimize the training set using KNN strategy to reduce the class imbalance
4. Calculate the statistical metrics of the predictor using the validation set.
5. Reiterate steps 2 to 5 ten times to obtain ten sets of statistical measures. Finally, compute the average of each metric.

We have conducted a 10-fold cross-validation scheme in this work, and the results are discussed in the following section.

## Results and Discussion

We designed a multilayer perceptron in Weka for the classification of phosphoglycerylated lysine residues. From the eight structural properties, we represented each lysine residue by a 40-dimensional feature vector. These eight structural properties were then investigated to deduce its importance for the classification process.

Every proposed predictor needs to be assessed thoroughly to find out about its performance. The subsections below discuss the target cross-validation scheme which we have used and the classification results of the multilayer perceptron.

### Target cross-validation

When using data-balancing approach, instead of the general jackknife test or general 10-fold cross-validation method, we have to use the so-called “target jackknife” or “target cross-validation” approach as done in^21,42,71^. This is because the balanced benchmark dataset can be only used to train the model and the test must target all the experimentally confirmed samples which are excluded in training. In our work, the benchmark dataset was obtained after filtering out the negative samples from a class imbalance ratio of 1:29 to 1:3. Before optimizing the benchmark dataset, the data samples were divided into 10 parts of about the same size. From the 10 sets, one set was singled out as a test dataset, and the remaining 9 were selected as the training dataset. The training set was then optimized to reduce the class imbalance to a ratio of 1:2 using KNN strategy as described in the “Validation Scheme” section.

### Comparison with the existing methods

The three recently proposed methods which we have compared our predictor to are iPGK-PseAAC predictor^38^, CKSAAP_PhoglySite method^15^ and Phogly-PseAAC predictor^36^. iPGK-PseAAC and Phogly-PseAAC predictors have a web-server to which we uploaded all of the protein sequences in the FASTA format to identify phosphoglycerylated lysine residues. It is important to note that the web-servers may have been trained with some of the protein sequences contained in sequences for performance evaluation and hence the result of these methods can be biased in their favor. For comparison with the CKSAAP_PhoglySite method, we constructed the features for lysine residues using their technique and trained similar classifier as ours to identify the phosphoglycerylated sites. For these three methods as well as ours, the performance was calculated on the validation set (the set of samples which were put aside for testing in the 10-fold cross-validation scheme).

**Figure 4:**
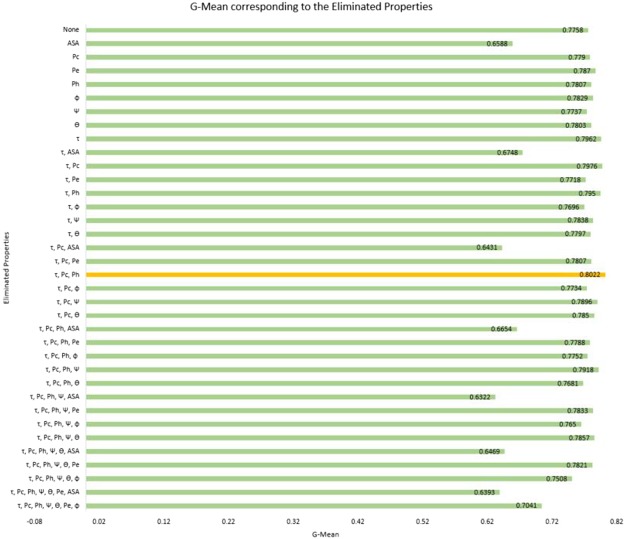
Graph showing G-Mean for the eliminated structural properties.

**Figure 5:**
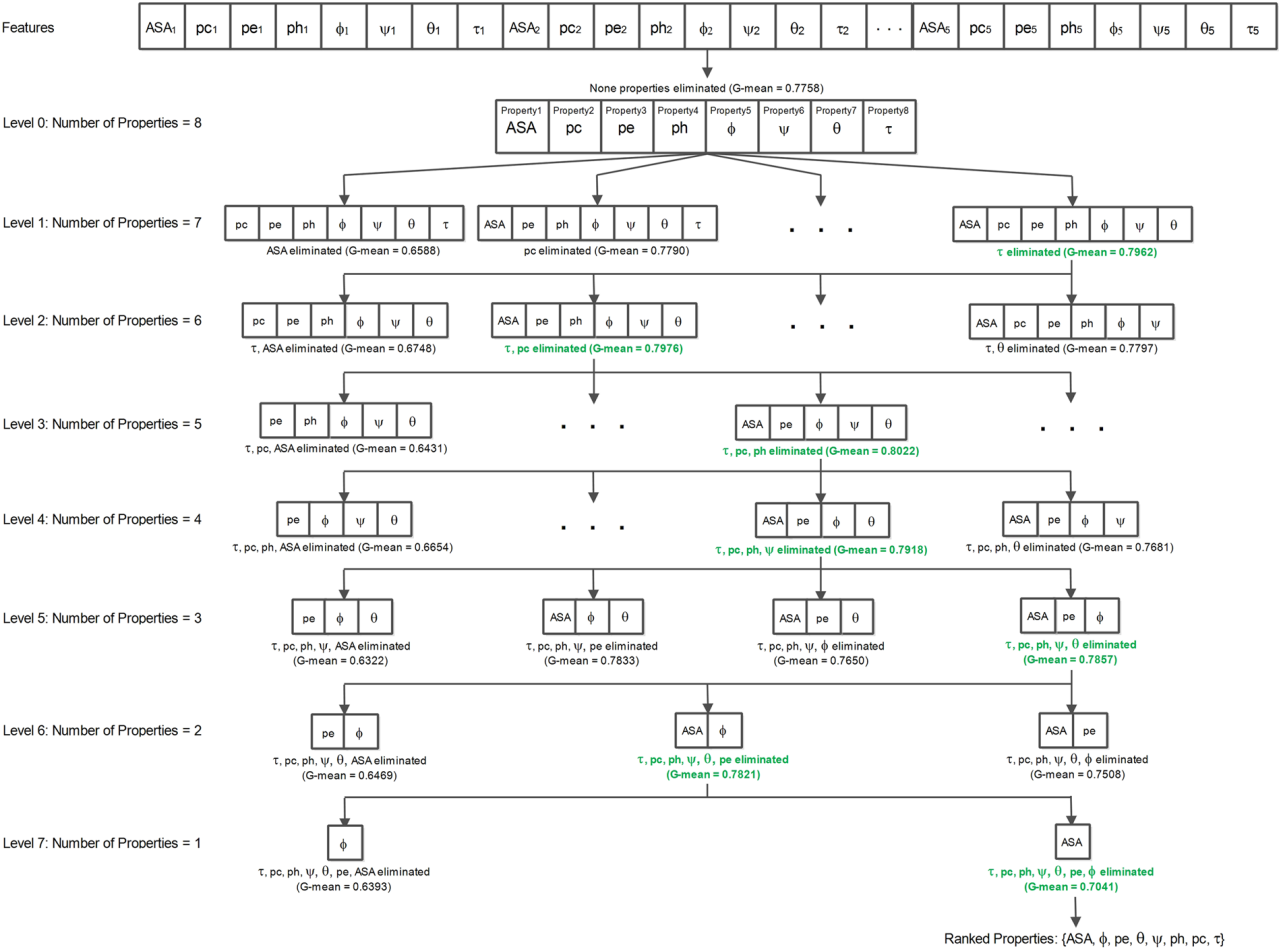
Backward elimination scheme performed on the eight structural properties.

We show the comparison of the iPGK-PseAAC predictor^38^, CKSAAP_PhoglySite method^15^, Phogly-PseAAC predictor^36^ and PhoglyStruct predictor in Table 1. As it can be seen, PhoglyStruct outperforms the three previous methods in the four metrics sensitivity, G-Mean, F-Measure, and AUC. This performance of PhoglyStruct is achieved using the backward elimination scheme^39^ when one of the torsion angles, *τ*, and two secondary structure properties, coil (*pc*) and helix (*ph*), are eliminated. Figure 4 shows the performance after applying the 10-fold cross-validation procedure while properties are successively eliminated. It can be seen from the figure that the highest performance (G-Mean = 0.8022) was achieved when the properties*τ*, *pc* and *ph* were eliminated from the feature set. The backward elimination of this work is portrayed in Fig. 5. The ranked properties obtained after completion of the feature selection process are {ASA, *ϕ*, *pe*, *θ*, *ψ*, *ph*, *pc*, *τ*}, where ASA is the most important property while *τ* is the least significant. The best performance was achieved when each lysine residue was represented by a 25-dimensional feature vector corresponding to properties such as *ASA, pe, ϕ, ψ*, and *θ*.

**Table 1:** Evaluation of the three benchmark prediction methods and PhoglyStruct predictor using the 10-fold cross-validation procedure. Metric with the highest value is highlighted in bold.

Method	Sensitivity	Specificity	G-Mean	Accuracy	MCC	F-Measure	AUC
iPGK-PseAAC^38^	0.4647	**0.9912**	0.6720	**0.8594**	**0.5950**	0.6136	0.7253
CKSAAP_PhoglySite^15^	0.4188	0.8992	0.602	0.7791	0.3638	0.4748	0.6568
Phogly-PseAAC^36^	0.6985	0.7809	0.7332	0.7592	0.4479	0.5921	0.7371
PhoglyStruct	**0.8542**	0.7597	**0.8022**	0.7834	0.5468	**0.6603**	**0.8077**

According to Table 1, sensitivity was improved significantly by 22.3%, followed by AUC 9.6%, G-Mean 9.4%, and F-Measure by 7.6%. These results show a substantial improvement over the previous prediction methods.

From the results, it can be seen that PhoglyStruct has shown promising performance. This can be attributed to the use of essential structural properties of proteins, which include accessible surface area (ASA), local structure conformation (*pe)* and backbone torsion angles (*ϕ, ψ,* and *θ*). ASA property can be seen from Figs 4 and 5 to be an important attribute as its absence significantly reduces the G-Mean. These characteristics of amino acids were computed using the SPIDER2 toolbox^44^ and have proved to be extremely useful in identifying the phosphoglycerylated lysine residues. The efficacy of structural properties has also been evident in areas such as MoRF detection^55^, subcellular localization of proteins^72^, protein fold recognition^54,73^ and so on. Performance of the classifier can be further improved by employing the feature selection techniques which have been proven to be a useful tool for classification and prediction problems in the area of proteomics^36,74–78^.

As pointed out in the article^79^ and indicated in a series of recent publications^38,80–89^, user-friendly and publically accessible web-servers represent the future direction for developing practically more useful prediction methods and enhance their impact^90,91^, we shall make efforts to establish the web-server for the prediction method represented in this paper.

## Conclusion

This paper introduces a new predictor called PhoglyStruct which makes use of the structural characteristics of proteins to identify phosphoglycerylated lysine sites. The structural properties such as accessible surface area, amino acid contribution to local structure conformation and backbone torsion angles have demonstrated to be important in distinguishing the phosphoglycerylated and non-phosphoglycerylated lysine residues. The issue of class imbalance was successfully solved by employing the k-nearest neighbors cleaning treatment. A balanced dataset alongside a multilayer perceptron classifier showed a considerable improvement in performance over the available predictors in the literature.

## Electronic supplementary material

supplementary materials
