# Supplementary material for: PhoglyStruct: Prediction of phosphoglycerylated lysine residues using structural properties of amino acids
Source: Sci Rep. 2018 Dec 18;8:17923. doi: 10.1038/s41598-018-36203-8 (PMC6299098; doi:10.1038/s41598-018-36203-8)
Supplement: Supplementary file 1 — supplementary materials [file 41598_2018_36203_MOESM1_ESM.pdf]

PhoglyStruct: Prediction of phosphoglycerylated lysine residues using structural properties of amino acids

Abel Chandra[5, *, †], Alok Sharma[1,2, 3,5,9 *, †], Abdollah Dehzangi[4], Shoba Ranganathan[6], Anjeela Jokhan[7], Kuo-Chen Chou[8,&], Tatsuhiko Tsunoda[2, 3, 9,&]

[†]Correspondence: abelavit@gmail.com; alok.sharma@griffith.edu.au
*Equal Contributors
[&]Last Authors
[1]Institute for Integrated and Intelligent Systems, Griffith University, Brisbane, QLD-4111, Australia.
[2]Department of Medical Science Mathematics, Medical Research Institute, Tokyo Medical and Dental University, Tokyo, Japan
[3]Laboratory for Medical Science Mathematics, RIKEN Center for Integrative Medical Sciences, Yokohama, Kanagawa, Japan
[4]Department of Computer Science, Morgan State University, Baltimore, Maryland, USA
[5]School of Engineering and Physics, Faculty of Science, Technology and Environment, University of the South Pacific, Suva, Fiji Islands
[6]Department of Molecular Sciences, Macquarie University, Sydney, NSW 2109, Australia
[7]Faculty of Science, Technology and Environment, University of the South Pacific, Suva, Fiji Islands
[8]The Gordon Life Science Institute, Boston, MA 02478, USA
[9]CREST, JST, Tokyo 113-8510, Japan

Supplement 1: Geometric Means for the different window sizes

	Upstream Downstream of lysine														
	±1	±2	±3	±4	±5	±6	±7	±8	±9	±10	±11	±12	±13	±14	±15
Sensitivity	0.787	0.8442	0.7211	0.7814	0.7959	0.7515	0.8455	0.7208	0.8009	0.7621	0.7159	0.7223	0.7673	0.6926	0.6309
Specificity	0.7135	0.7148	0.7644	0.751	0.6996	0.7049	0.664	0.7395	0.7441	0.7232	0.7372	0.7554	0.7424	0.7461	0.7644
Accuracy	0.7302	0.7475	0.7529	0.7565	0.7239	0.7162	0.709	0.7351	0.7589	0.7331	0.7333	0.7461	0.7481	0.7338	0.7335
MCC	0.446	0.49	0.4463	0.4785	0.447	0.402	0.448	0.414	0.488	0.432	0.406	0.431	0.455	0.399	0.372
G-Mean	0.7493	0.7758	0.7424	0.7660	0.7462	0.7278	0.7493	0.7301	0.772	0.7424	0.7265	0.7387	0.7547	0.7189	0.6944

Supplement 2: Comparison of PhoglyStruct with simpler set of features

The table below shows the comparison of PhoglyStruct with simpler set of features for the same 10-fold cross-validation set. Simpler_Features are features for each lysine taking into account the occurrence of amino acids in the ±2 residue window. The matrix representing each lysine is of the size 20x5 where 20 is the different types of amino acid in the genome and 5 is the 2 upstream, the lysine and 2 downstream amino acid. Out of the 20 amino acids, a value of 1 is given to the amino acid present at that position otherwise the rest are given a value of 0. Therefore, the feature representing each lysine is a 100-dimensional vector. As it can be seen, PhoglyStruct, which uses the structural properties of amino acids, outperforms this simpler set of features.

Method	Sensitivity	Specificity	G-Mean	Accuracy	MCC	F-Measure	AUC
Simpler_Features	0.6470	**0.7490**	0.6887	0.7233	0.3612	0.5326	0.6982
PhoglyStruct	**0.8726**	0.7331	**0.7976**	**0.7678**	**0.5343**	**0.6513**	**0.8034**
